# Erythropoiesis and Iron Homeostasis in Non-Transfusion-Dependent Thalassemia Patients with Extramedullary Hematopoiesis

**DOI:** 10.1155/2019/4504302

**Published:** 2019-01-30

**Authors:** Yumei Huang, Rongrong Liu, Xiaoyun Wei, Jiaodi Liu, Lingyuan Pan, Gaohui Yang, Yongrong Lai

**Affiliations:** Department of Hematology, The First Affiliated Hospital of Guangxi Medical University, Nanning, China

## Abstract

**Background:**

Extramedullary hematopoiesis (EMH) is common in non-transfusion-dependent thalassemia (NTDT) patients. Clinical presentations of EMH vary as MRI screening is not feasible. Hence, serum biomarkers are used to predict the risk of EMH.

**Materials and Methods:**

52 NTDT patients, including 26 EMH (+) and 26 EMH (-), together with 26 healthy controls, were enrolled in this case-control study from 2013 to 2016. EMH was confirmed by computed tomography or MRI. Demographic, transfusion, genetic, laboratory, and liver iron concentration (LIC) data, as well as clinical complications, were analyzed.

**Results:**

EMH (+) patients had significantly higher serum ferritin (SF), growth differentiation factor 15 (GDF15), and erythropoietin (EPO) levels compared with EMH (-) patients and controls. The levels of erythroferrone (ERFE), hepcidin, and sTfR did not differ significantly between EMH (+) and EMH (-) patients (p>0.05). In NTDT patients, serum ERFE was not related to SF, LIC, hepcidin, sTfR, EPO, GDF15, and Hb levels. GDF15, EPO concentrations, and GDF15 to sTfR and GDF15 to EPO ratios are able to determine the presence of EMH with considerable sensitivity and specificity.

**Conclusions:**

GDF15, EPO, and GDF15 to EPO and GDF15 to sTfR ratios are potential biomarkers for the early prediction of NTDT in patients who are at risk for EMH.

## 1. Introduction

In contrast to transfusion-dependent thalassemia (TDT) which requires regular and ongoing lifelong transfusions for survival, non-transfusion-dependent thalassemia (NTDT) is defined as a disease state not requiring regular transfusion, although occasional or even frequent transfusions under certain clinical situations may be accepted for a restricted period of time. NTDT includes *α*-thalassemia intermedia (hemoglobin H disease), *β*- thalassemia intermedia, and mild/moderate hemoglobin E/*β*-thalassemia [1]. Following birth, extra-medullary hematopoiesis (EMH) is the proliferation and expansion of blood forming tissues outside the typical anatomic locations, in order to compensate for physiologically defunct erythrocytes insufficient which cannot meet the demands of a normal circulatory system. This uncontrolled expansion of early erythroid progenitors can lead to reduced bone marrow function and increased erythropoiesis as well as erythrocytes which have impaired oxygen exchange capacity [2]. EMH is particularly common in NTDT patients (20%) but rarely occurs in patients with TDT (<1%) who also exhibit underlying bone marrow suppression due to transfusion therapy [2, 3]. No detailed demographic data on Chinese NTDT patients with EMH are available currently.

The clinical presentation of EMH varies and is determined by the extent and anatomical location, such as spleen, liver, lymph nodes, thymus, heart, breasts, prostate, broad ligaments, kidneys, adrenal glands, pleura, retroperitoneal tissue, skin, peripheral and cranial nerves, and the spinal canal [4]. Although different anatomical locations may be involved, paraspinal involvement (which occurs in 11%–15% of cases) attracts the most attention because of the potentially debilitating permanent clinical consequences which are secondary to spinal cord compression [4]. Therefore, early diagnosis of EMH may impact on the course of disease management and thus may reduce the incidence of irreversible neurologic damage. EMH diagnosis depends primarily on imaging; specifically, magnetic resonance imaging (MRI) has become the method of choice for diagnosis, localization and measurement of EMH and follow-up evaluation for spinal and paraspinal pathology [4]. It is conceivable that early detection of paraspinal EMH might prevent cord compression with permanent neurological damage. However, due to the costs, access to MRI is often restricted for thalassemia patients as the majority of them do not live in first tier countries. Moreover, no guidelines are in place as to how to utilize MRI in the asymptomatic patients, specifically as a screening procedure for paraspinal EMH. Only patients with obvious clinical manifestations or core compression were indicated to be screened by MRI; furthermore, patients at risk for EMH would need to be scanned periodically, with possible scanning of larger anatomical areas. In summary, we feel strongly that the availability of a reliable biomarker which identifies patients with EMH would be of great benefit to these patients.

Growth differentiation factor 15 (GFD15) is elevated in patients with ineffective erythropoiesis and therefore might be a suitable candidate marker for patients with EMH. We hypothesized that GDF15 levels would correlate with the degree of impaired erythropoiesis and also its sequential clinical features [5]. Anemia as well as hypoxia leads to a compensatory increase in serum erythropoietin (EPO) levels to compensate for reduced end organ oxygen delivery. The marked increase in EPO stimulation can result in uncontrolled expansion of erythroid precursors in the bone marrow and other sites, leading to EMH [6]. Recently, a prospective study of adult NTDT patients demonstrated a close relationship between the soluble form of transferrin receptor (sTfR) and the EMH, which may help predict the presence of EMH, particularly in patients with spleen involvement [7]. Erythroferrone (ERFE) is a novel hormone recently reported by Ganz et al., which is a member of the C1q-tumor necrosis factor-related family of proteins. ERFE is produced by erythroid precursors in response to EPO and negatively regulates liver hepcidin production during stress or ineffective erythropoiesis [8]. Drakesmith et al. showed that ERFE suppresses hepcidin by inhibiting hepatic BMP/SMAD signaling by impairing members of the BMP5, BMP6, and BMP7 subgroup of BMPs, indicating that ERFE can act as a natural ligand trap generated by stimulated erythropoiesis to regulate the availability of iron. Therefore it is important to measure the serum ERFE levels in thalassemia patients particularly in those with EMH, to clarify evidence of the role of ERFE in ineffective erythropoiesis [9]. ERFE levels were only analyzed by Swinkels et al. and Ganz et al. in *β* thalassemia patients and blood donors [10, 11]. These observations support the hypothesis that GDF15, EPO, sTfR, and ERFE may not only be associated with EMH but could possibly serve as predictive biomarkers for EMH. Thus far, no conclusive or prospective data have been reported for NTDT patients.

The aim of this study in NTDT patients was to establish relationships between the erythropoiesis biomarkers (GDF15, EPO, sTfR, and ERFE) and iron overload markers (SF, LIC, and hepcidin) with the severity as well as complications of EMH. We hypothesized that the results could potentially facilitate establishing these markers as predictive biomarkers for the risk of developing EMH and its complications.

## 2. Materials and Methods

In this case-control study, 52 patients with NTDT (26 patients with EMH and 26 without) were enrolled in the First Affiliated Hospital of Guangxi Medical University, between June 2013 and June 2016. All patients were diagnosed with NTDT based on previously described criteria [1]. Before enrollment EMH was confirmed or excluded by computed tomography (CT) or MRI for preoperative examination, evaluation of respiratory function, assessment of pathological abdominal and mediastinal lymphadenopathy, neurological or orthopedic dysfunction evaluation, and other clinical reasons. The 52 NTDT patients were age, sex, and thalassemia-type matched. In addition, 26 age and sex matched healthy subjects without anemia or liver disease were enrolled as a control group who were recruited from the Medical Examination Center of the First Affiliated Hospital of Guangxi Medical University. Signed informed consent was obtained from all subjects or their parents (for those less than 18 years old). This study was approved by the Medical Ethics Committee of the First Affiliated Hospital of Guangxi Medical University (approval number: 2015(KY-E-031)). Patient demographic data as well as disease specific historic data were obtained retrospectively.

Serum samples were cryopreserved at -80°C for batched analysis at a later time point. Hematological parameters, SF levels, hemoglobin type and quantitation (HbA, HbA2, and HbF levels), and other reported biochemical parameters were all analyzed at the clinical laboratory of the First Affiliated Hospital of Guangxi Medical University. A total of 338 known mutations linked to *α*- or *β*-thalassemia were genotyped by the Huada Gene Medical Institute for each enrolled thalassemia patient.

Serum hepcidin concentrations were measured by enzyme linked immunosorbent assay (ELISA) (Bachem Group, CA USA, Lot No. A15840); serum sTfR and GDF15 amounts were determined by ELISA with specific kits (R&D Systems, Minneapolis, MN USA, Lot Nos. 339307 and 338342 respectively). EPO levels were measured by ELISA (eBioscience, Vienna, Austria, Lot No. 124052015). ERFE concentrations were measured by the Human Protein FAM132B ELISA Kit (My BioSource, San Diego, Ca, USA, Lot No. R09144170). All assays were carried out according to the manufacturer's instructions.

Liver iron concentration (LIC) measurements were performed by MRI analysis (Ferriscan®-Resonance Health, Australia) and myocardial iron deposition was assessed as previously described by our group [12].

## 3. Statistical Analysis

Data were analyzed with the SPSS software (Version 16.0; SPSS Inc., USA). Mean ± SD or median (range) and frequency and percentages were used to describe continuous and categorical variables, respectively. Independent samples t-test (or Mann–Whitney U-test) and chi-squared test (or exact test) were used to compare continuous and categorical variables, respectively, between two groups; one-way ANOVA was employed for three groups. Bivariate correlation analysis was performed with Pearson or Spearman correlation. P<0.05 was considered statistically significant. Area under the receiver operating characteristic (ROC) curve was used to determine whether any level of erythropoiesis biomarkers could differentiate between patients with and without EMH; z-test was used to compare areas under ROC curves.

## 4. Results

26 patients with confirmed EMH were identified, with a median age of 27.5 (19-57) years, of whom 57.7% were males. The median age of matched patients without EMH was 27.5 (17-63) years, with 61.5% being males. Healthy controls were 28 (18-57) years old, with 57.7% being males.

The patient characteristics and clinical parameters are summarized in Table 1. EMH (–) patients were older at the onset of anemia compared with their EMH (+) counterparts (6.0 vs 3.0 years, p=0.013). Anemic heart disease is defined according the echocardiogram results and was characterized by cardiac enlargement, cardiac hypertrophy, or cardiac dysfunction. The proportion of patients with splenectomy and anemic heart disease were similar in both groups. EMH (+) patients had a higher incidence of cholelithiasis compared to EMH (–) patients (53.8% vs 15.4%, p=0.028). No patient was diagnosed with diabetes and the percentages of patients with impaired glucose tolerance and elevated parathyroid hormone were similar in both groups. One EMH (+) patient had hypothyroidism and one EMH (-) patient had hyperthyroidism and three subclinical hypothyroidism.

The analysis for erythropoiesis and iron metabolism biomarkers is shown in Table 2. Hb levels were significantly different among the three groups; the Hb levels in healthy controls were higher than those of NTDT patients, although EMH (+) patients had lower Hb levels than the EMH (-) group; the difference was not statistically significant. Although as expected HbF and HbA2 levels in NTDT patients were higher than those of healthy controls, the difference between EMH (+) and EMH (-) patients was not statistically significant (p =0.36 and p=0.087, respectively).

Chronic iron overload was assessed by SF, LIC, and cardiac T2*∗* values; hepatocellular dysfunction was determined by alanine aminotransferase (ALT) and aspartate transaminase (AST) levels. Healthy controls showed no LIC and cardiac T2*∗*. EMH (+) patients displayed the highest ALT levels compared to EMH (-) and healthy controls (26.5 vs 22 vs 17 U/L, p=0.001, respectively), and EMH (+) and EMH (-) patients showed significantly higher AST levels than healthy controls (34 vs 32 vs 16.5 U/L, p=0.001 respectively). AST and ALT levels were similar in EMH (+) and EMH (-) patients. Median SF levels were significantly different among the three groups, with EMH (+) patients displaying the highest levels compared to EMH (-) and health controls (2450.5 vs 1458.0 vs 138.05 ng/mL, p<0.001, respectively). The difference seen in EMH (+) and EMH (-) patients reached statistical significance (p<0.001), and 50.0% of patients in the EMH (+) group had SF levels in excess of 2500ng/mL compared to 15.4% in the EMH (-) group. Although LIC levels were higher in the EMH (+) group than in the EMH (-) group, they did not reach statistical significance. Mean Cardiac T2*∗* values (ms) were not significantly different between the EMH (+) and EMH (-) groups. Taken together, almost all NTDT patients had liver iron overload but no cardiac iron overload.

In order to establish a correlation between erythropoiesis and iron metabolism, serum levels of hepcidin, EPO, GDF15, sTfR, and ERFE were measured. All indexes showed significant differences between NTDT patients (EMH (+) and EMH (-) patients) and normal controls; however, only GDF15 and EPO levels in EMH (+) patients also showed significantly higher levels when compared to the EMH (-) group (GDF15 9428.32 vs 3097.80 vs 176.02, p<0.001 and EPO 30.0 vs 40.62 vs 5.85, p<0.001, respectively).

**Figure 1:**
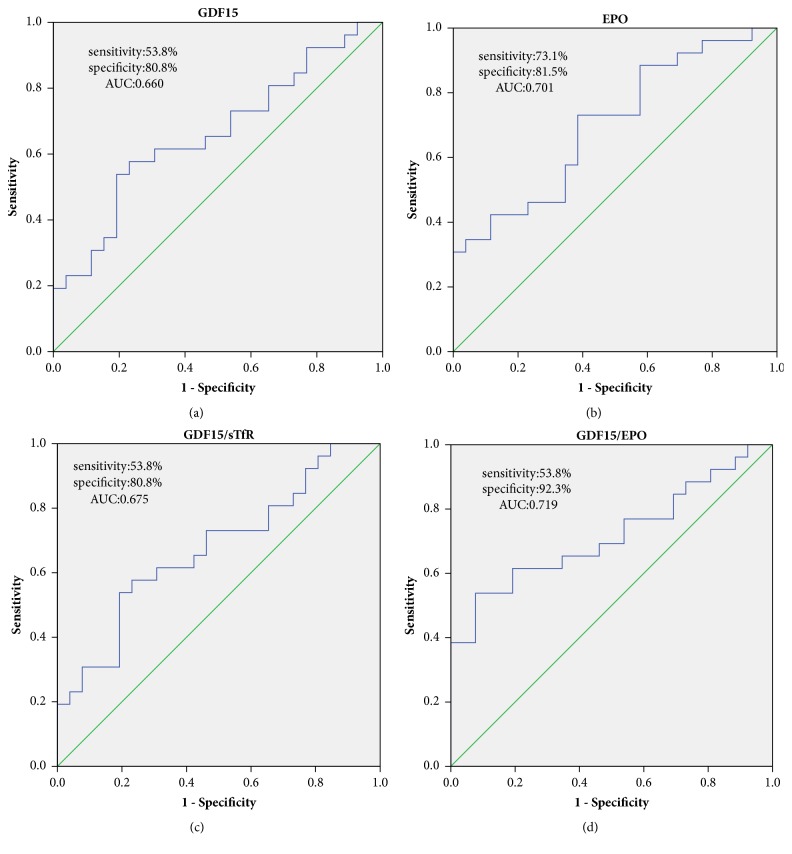
ROC curve analysis of (a) GDF15, (b) EPO, (c) GDF15/sTfR ratio, and (d) GDF15/EPO ratio as biomarkers for differentiating EMH (+) from EMH (-) patients.

Correlative results for GDF15, ERFE, and iron metabolism and erythropoiesis markers in NTDT patients are summarized in Table 3. GDF15 demonstrated a weak to intermediate correlation with biomarkers of erythropoiesis and iron metabolism; it was associated positively with SF (r=0.298, P=0.036)), LIC (r=0.375, p=0.009)), and sTfR (r=0.384, p=0.005). However, it was correlated inversely with hepcidin (r=-0.347, p=0.012) in NTDT patients. We found only GDF15 to be inversely correlated with hepcidin (r=-0.413, p<0.05), positively associated with EPO (r=0.516, p<0.05), and inversely correlated with hepcidin /ERFE ratios (-0.458, p<0.05) in EMH (+) patients. Next, AUC_ROC_ was used to determine whether any of the biomarkers could differentiate patients with and without EMH. GDF15 and EPO concentrations and GDF15 to sTfR and GDF15 to EPO ratios were able to determine the presence of EMH with considerable sensitivity and specificity (Figure 1). The predictive effects of the four biomarkers were not significantly different according to the AUC_ROC_ values.

Between EMH (+) patients, the anatomical locations for the extra-medullary erythropoiesis were variable.  Table 4 summarizes the anatomical EMH regions. Besides common EMH sites such as thoracic-dorsal, lumbar and sacral regions, atypical EMH locations included cerebral flax, lesser omentum, postcava, portal vein, and left renal vein.

## 5. Discussion

EMH, as a physiological compensatory mechanism resulting from ineffective erythropoiesis, is usually found incidentally. Thus far its diagnosis has not been correlated to any specific clinical or biochemical parameter [7]. However, the availability of correlative or predictive markers could enhance clinical care of some patients with thalassemia.

In our study, younger patients with EMH (+) and a more aggressive clinical course and earlier onset of anemia and elevated ferritin levels were enrolled. In contrast, the cohort reported by Ricchi et al. consisted of EMH (+) patients who were significantly older and many had started transfusion and chelation therapies [13]. We believe the differences seen in our study may be due to EMH being related to chronic anemia and ineffective erythropoiesis, while in TDT patients optimal transfusion therapy reduces erythroid marrow stimulation and expansion [14].

GDF15, a member of the transforming growth factor-*β* (TGF-*β*) superfamily of cytokines and considered a marker of ineffective erythropoiesis, might be indicative of ineffective erythropoiesis and iron overload, two conditions normally found in NTDT patients. In this study, we found GDF15 levels in the EMH (+) group of patients to be elevated significantly over levels in EMH (-) patients. In our study we found a highly significant correlation for GDF15 to be an ineffective erythropoietic response marker, corroborating previous findings that GDF15 levels are elevated significantly in patients with ineffective erythropoiesis and associated with clinical severity in NTDT patients [5, 15, 16]. GDF15 may have utility as a biomarker for a variety of diseases and may also identify NTDT patients at increased risk of pulmonary and cardiovascular complications as well as subclinical atherosclerosis [17]. This study shows that GDF15 levels might be considered a biomarker to predict EMH according to the ROC curve analysis data.

EPO is a renal glycoprotein that functions as the main erythropoiesis regulator, both under basal and stress conditions. Increased EPO synthesis is also reflected by marrow expansion [6]. In this study, the EPO levels were significantly higher in NTDT patients than in healthy controls. However, the possible reason for the relative lower levels in EMH (+) group of patients than in the EMH (-) group, maybe that the transfusion requirement of EMH (+) patients was higher, which partly suppressed the ineffective erythropoiesis, thus leading to relatively lower EPO levels. In this study, log_GDF15_ levels were positively correlated with log_EPO_ levels, although only in EMH (+) cases. These findings indicate that the GDF15/EPO ratio may also differentiate patients with and without EMH, similar to GDF15 or EPO alone, or the GDF15/sTfR ratio, according to the ROC curve analysis data.

sTfR is a biomarker of erythropoiesis. When SF levels are normal or high, sTfR levels reflect the erythropoietic activity [15]. Previous studies showed that, in NTDT patients, sTfR amounts could represent a predictive factor for EMH, particularly in NTDT patients with a spleen [7]. In our study, however, the difference in sTfR levels between EMH (+) and EMH (-) groups was not significant, and the protein could not predict EMH either. This discrepancy may be due to the fact that splenectomy is considered a risk factor for paraspinal EMH. This further supports the hypothesis of an association between iron metabolism and erythropoiesis expansion as well as the impact of splenectomy on EMH [6, 7].

Splenectomy, which is associated with an increase of sTfR, may adversely affect the degree of ineffective erythropoiesis, thus modifying GDF15 levels and thus far unknown “erythroid regulators” which increase the risk for EMH and iron overload [6, 18]. However, the number of splenectomized patients was evenly distributed in both groups. It was suggested that GDF15 might be more specific for ineffective erythropoiesis, while sTfR could be considered a more general marker of erythropoietic activity [15, 19]. However, it is possible that this discrepancy may reflect differences in patient ethnicity, environmental factors, or it could be due to the small sample size of this study.

Erythropoiesis and iron metabolism are closely interconnected. Indeed, erythropoiesis participates in systemic iron homeostasis by regulating hepcidin, which is upregulated in response to iron overload and inflammation and downregulated by erythropoietic stimuli such as anemia, hypoxia, and EPO synthesis/administration [6]. In thalassemia, ineffective erythropoiesis induces the release of factors including GDF15, twisted gastrulation protein homolog 1 (TWSG1), hypoxia-inducible factor (HIF), and ERFE, which can all inhibit hepcidin release [20]. A negative correlation of GFD15 with hepcidin corroborates the suppression of the iron regulatory protein and hepcidin, which is consistent with our findings and also applicable in NTDT patients. Tanno et al. reported that individuals with *β*-thalassemia have elevated serum GDF15 levels which are positively correlated with sTfR, EPO, and SF levels and negatively associated with hemoglobin levels [18]. These data suggest that excessive release of GFD15 may suppress hepcidin production and thus dysregulate iron homeostasis in thalassemia syndromes [18, 20].

We found that GDF15 was only negatively correlated with hepcidin in the EMH (+) group of patients. This might indicate that high GDF15 expression levels found in states of ineffective erythropoiesis may contribute to pathological iron overload through a mechanism of incomplete hepcidin suppression. These findings also suggest that GDF15 is not the sole regulator of hepcidin expression but instead contributes to hepcidin suppression in the pathological setting of thalassemia. Hepcidin deficiency which is mediated by GDF15 overexpression arising from an expanded erythroid compartment probably contributes to iron overload in thalassemia patients by inhibiting hepcidin expression and subsequently participates in tissue iron overloading in these patients [18, 21]. In our study, the hepcidin was relatively higher in EMH (+) patients compared to EMH (-) patients, but this did not reach statistical significance. Increased transfusion requirements in EMH (+) patients may have led to an appropriate response to increase hepcidin in response to relative iron overload, which perhaps explains why there are no differences in LIC and heart iron levels as seen by MRI scans.

A mouse model of TI indicated that ERFE could be a pathological suppressor of hepcidin in ineffective erythropoiesis [22]. The plasma ERFE concentrations in *β*-TI patients in Swinkels et al. paper and serum ERFE concentrations in nontransfused and pretransfusion thalassemia patients in Ganz paper were significantly higher relative to blood donors, which is different from ours [10, 11]. The absence of a correlation between serum ERFE levels and iron metabolism or erythropoiesis markers in NTDT patients in our study is similar to a previous report by Swinkels et al. from blood donors and *β*-TI patients, demonstrating that plasma ERFE concentrations were independent of EPO and hepcidin concentrations [10]. However, in contrast to our observations, a correlation between ERFE and biomarkers of erythropoiesis and iron metabolism was reported in hemodialysis patients [23]. The absence of a correlation in our study may be due to the delicate nature of ERFE which is prone to degradation due to temperature changes such as freeze thawing. Alternatively, the discrepancy could be due to the small sample size of our study impacting on the statistical analysis. However, in Ganz paper hepcidin concentrations inversely correlated with ERFE concentrations, consistent with the proposed role of ERFE as a pathological hepcidin suppressor in *β*-thalassemia. This strongly suggests that the assay used in the Swinkels paper and our paper does not represent true human ERFE levels. We suggest that standardization of the reference antigen and the development of more reliable laboratory reference ranges in multicenter of thalassemia patients and healthy adults and children are needed [11].

In EMH (+) patients, the correlation between ERFE and sTfR differed from that in hemodialysis patients, and the negative correlation between hepcidin/ERFE and GDF15 suggests that the ERFE/hepcidin pathway may play a role in NTDT patients with EMH (+). Currently, there is no evidence showing that ERFE can regulate sTfR and GDF15 directly. Nevertheless, we are planning to investigate the role in ERFE production and conversely the role of ERFE in sTfR and GDF15 expression at the erythroblast level.

In NTDT patients, ineffective erythropoiesis and peripheral hemolysis are the central processes leading to inappropriately low levels of hepcidin and increased intestinal iron absorption [1, 15]. We believe the elevated ferritin levels in our study support this hypothesis. Furthermore, we believe the small sample size resulted in failure to demonstrate a statistical difference between LIC and T2 and the iron metabolism markers (hepcidin and ERFE) between EMH (+) and EMH (-) patients. Moreover, the GDF15 and EPO levels were higher in EMH (+) patients compared to EMH (-) patients. The relationship between EMH and iron metabolism remains controversial in TDT patients. Ricchi et al. showed that EMH was associated with a lower cardiac iron load [13]. In contrast, Sousos et al. showed that EMH was not correlated with cardiac iron accumulation [24, 25]. No such relationship has been reported in NTDT patients and our data seem to suggest that an expanded erythropoietic pattern may affect iron distribution, although this requires clarification by further research.

EMH is a physiological compensatory phenomenon, which may involve a large number of sites all over the body. The thoracic and lumbar regions are most commonly involved in this process. In the current study, EMH was about 80% higher than reported previously (only 11–15% for paraspinal location in cases with EMH) [4, 26]. This may be explained by the fact that, with the availability of more scanners, more patients are undergoing CT scans, although certain anatomical locations are ignored because there is no targeted scanning for EMH. Some anatomical sites are thought to engage in active hematopoiesis during fetal life. However, this pathway normally stops at birth, but extra-medullary hematopoietic vascular connective tissues retain their ability to synthesize red blood cells in long-standing ineffective erythropoiesis [4].

In the absence of transfusion therapy, the pathophysiological mechanisms emanating from ineffective erythropoiesis and peripheral hemolysis can lead to a multitude of clinical complications in patients with NTDT [27]. An assessment of 584 TI patients indicated that older age and splenectomy are independently associated with an increased risk of most disease-related complications. In addition, splenectomy was shown to be a risk factor for paraspinal EMH, further supporting the notion of a close association between iron metabolism with erythropoiesis expansion, as well as the impact of splenectomy on their status [2, 7, 14]. Several studies have shown that transfusion dependent TM patients with EMH have no or low cardiac iron overload [13, 24]. In agreement with this study of Chinese NTDT patients with EMH, LIC levels do not significantly differ between EMH (+) and EMH (-) patients. However, it is reasonable to assume that iron overload in EMH (+) patients is more serious. Indeed, in an unpublished study of 158 NTDT patients, we found a median LIC of 8.9 mg/g dry weight of tissue.

The association of iron overload and target–endocrine gland toxicity has been established in *β*-thalassemia major in previous studies, and endocrinopathy in NTDT patients is relatively lower than in *β*-thalassemia major cases [2, 14, 27, 28]. In this study, the frequencies of endocrinopathy were not different; however, considering the higher rate of cholecystolithiasis in EMH (+) patients, the current findings confirmed the role of ineffective erythropoiesis and peripheral hemolysis in the progression of EMH.

## 6. Limitations

The limitations of this study were as follows: (1) patients were not enrolled continuously; (2) a small sample size was used due to the scarcity of EMH cases; (3) MRI scans could not be performed in all cases. Although the evaluation and radiographic as well as biomarker correlation of these patients was a snapshot at a defined time point in their disease, the data obtained justify moving forward with the implementation of a larger multicenter trial.

## 7. Conclusions

Overall, GDF15 and EPO levels were elevated in NTDT patients with EMH. The associations of GDF15 and EPO levels with erythropoiesis markers and iron overload support their potential value in early identification of NTDT patients with a risk of EMH. Further studies are needed to determine the practical usefulness of GDF15 and EPO for differentiating EMH (+) from EMH (-) cases.

## Figures and Tables

**Table 1:** Characteristics and clinical parameters of NTDT patients (with and without EMH) and normal controls.

**Parameters**	**EMH (+)**	**EMH (-)**	**Normal**	**p value**
	**n= 26**	**n= 26**	**n=26**	
**Female N (**%**)**	15 (57.7%)	16 (61.5%)	15 (57.7%)	0.95
**Age (years)**	27.5 (19-57)	27.5 (17-63)	28 (18-57)	0.77
**age <20**	2 (7.7%)	3(11.5%)	3(11.5%)	0.82
**20≤ age<35**	16 (61.5%)	16 (61.5%)	15 (57.7%)	
**age ≥35**	8 (30.8%)	7 (26.9%)	8 (30.8%)	
**Age of onset (years)**	3 (0.5-32)	6.0 (1-24)	NA	0.013
**Thalassemia type**				0.85
**HbH disease **	8 (30.8%)	9 (34.60%)	NA	
β**-thalassemia intermedia**	11 (42.3%)	10 (38.5)%)	NA	
**Moderate hemoglobin E/**β**-thalassemia**	7 (26.9%)	7 (26.9%)	NA	
**Splenectomy N (**%**)**	16 (61.5%)	13 (50.0%)	NA	0.40
**Cholelithiasis**	14 (53.8%)	4 (15.4%)	NA	0.028
**Anemic heart disease**	5 (38.5%)	4 (17.9%)	NA	0.16
**Impaired glucose tolerance**	5 (19.2%)	4 (15.4%)	NA	1.00
**Elevated parathyroid hormone**	4 (15.4%)	6 (23.1%)	NA	0.74
**Median number of lifetime transfusion(ml/year)**	25.93(0-2488.87)	17.67(0-318.18)	NA	0.35
**Previous transfusion**				
**Yes**	21 (80.8%)	14 (53.8%)	NA	0.039
**No**	5 (19.2%)	12 (46.2%)	NA	

**Table 2:** Biomarkers of erythropoiesis and iron metabolism of NTDT patients with and without EMH and normal controls.

**Parameters**	**EMH (+)**	**EMH (-)**	**Normal**	**p value**
	n=26	n=26	n=26	
Hb(g/L)	71.5±15.11^★^	75±14.68^★^	136.96±12.39	<0.001
Hb<60	5(19.2%)	2(7.7%)		0.226
60≤Hb<90	15(57.7%)	17(65.4%)		
Hb≥90	6(23.1%)	7 (26.9%)		
A2	4.93(1.2-46.8)^★^	4(0.5-61.5)^★^	2.8 (2.2-3.3)	0.003
F	22.6(0.2-95.8)^★^	25.6(0.1-97.3)^★^	0.4 (0.2-1.2)	<0.001
Liver Function				
ALT(U/L)	26.5(4-80)^★^	22(7-65)	17(9-28)	0.001
AST(U/L)	34(12-122)^★^	32(11-119)^★^	16.5(7-35)	0.001
Serum ferritin(ng/mL)	2450.50(13.85-8300.0)^★,●^	1458.0(82.78-6128.0)^★^	138.05(30.90-535.7)	<0.001
SF<800	7 (26.9%)	6 (23.1%)		0.017
800≤SF<2500	6 (23.1%)	16 (61.5%)		
SF≥2500	13 (50.0%)	4 (15.4%)		
LIC (mg/g dry tissue)	20.05±14.74	17.85±12.43	NA	0.74
LIC<3	1(4.8%)	1(4.8%)		0.760
3≤LIC<7	6(25.0%)	5(20.8%)		
7≤LIC<15	5(20.8%)	5(20.8%)		
LIC>15	12(50.0%)	13(54.2%)		
Cardiac T2*∗*(ms)	33.38±15.18	31.59±14.33	NA	0.73
T2*∗*<20	2(15.3)	3 (13.0%)		0.846
T2*∗*≥20	11(84.6%)	20 (87.0%)		
Hepcidin ( ng/mL)	30.47(0.66-154.3)^★^	22.06(1.0-130.67)^★^	112.38(31.50-170.40)	<0.001
EPO( mIU/mL)	30.0(6.66-114.36)^★,●^	40.62(10.03-13472.0)^★^	5.85(3.5-29.40)	<0.001
GDF-15( pg/mL)	9428.32(536.28-38660.57)^★,●^	3097.8(6.95-34298.90)^★^	176.02(58.57-953.62)	<0.001
STfR( mg/L)	8.15 (2.42-8.47)^★^	8.07 (3.07-9.87)^★^	2.63 (0.32-5.34)	<0.001
GDF-15/hepcidin ratio	483.96 (6.04-4807.2)^★^	121.62 (0.21-34398.3)^★^	1.77(0.63-6.78)	<0.001
GDF-15/EPO ratio	310.86 (42.09-4609.2)^★,●^	83.04 (6.0-1002.3)^★^	27.08(3.89-138.85)	<0.001
GDF-15/sTfR ratio	1257.62 (94.8-4609.2)^★,●^	420.6 (0.87-4136.87)^★^	0.64(18.33-330.18)	<0.001
ERFE(ng/mL)	0.324(0.156-20.826)^★^	0.379(0.156-2.067)^★^	0.843(0.156-4.774)	0.027
Hepcidin/ERFE ratio	43.51(1.97-989.49)^★^	38.08 (3.13-524.26-0.319)^★^	136.06(14.90-963.76)	0.031
EPO/ERFE ratio	98.47(0.88-368.71)^★●^	150.03(27.61-33718.6)^★^	8.28(1.35-49.04)	0.003
ERFE/sTfR ratio	0.04(0.018-3.132)^★^	0.054(0.019-0.252)^★^	0.39(0.037-7.589	<0.001
ERFE/GDF15 ratio	0.00003(0.0000095-0.0055)^★●^	0.00007(0.0000069-0.256)^★^	0.0055(0.00029-0.025)	<0.001

Significant difference between EMH (+), EMH (-), and normal controls: ^★^significant difference when compared to normal controls; ^●^significant difference when compared to EMH (-).

**Table 3:** Correlation between GDF15 and ERFE values with iron metabolism and erythropoiesis parameters in patients with NTDT.

**Parameters **	**SF**	**LIC**	**Hepcidin**	**sTfR**	**EPO**	**Hb**	**GDF15**
**GDF15**	0.298(0.036)	0.375(0.009)	-0.347(0.012)	0.384(0.005)	-0.010(0.942)	0.124(0.057)	NA
**ERFE**	0.183(0.204)	0.250(0.087)	-0.185(0.188)	-0.110(0.439)	-0.011(0.938)	0.197(0.162)	0.124(0.382)

Correlation coefficients and significance (in brackets) are shown.

**Table 4:** Distribution and percentage of extra-medullary hematopoietic tissue.

**Location**	**N**	**percentage**
Parathoracic region	10	38.46%
Rib+thoracic+lumbar region	3	11.54%
Thoracic+rib	2	7.69%
Thoracolumbar region	2	7.69%
Rib	2	7.69%
Thoracic spinal canal+ lumbar and sacral canal	2	7.69%
Paramediastinum	1	3.85%
Rib+Thoracic spinal canal+thoracolumbar+sternum	1	3.85%
Rib+parathoracolumbar+lesser omentum + postcava+portal vein + left renal vein	1	3.85%
Cerebral falx+parathoracic+anterior sacral	1	3.85%
Parathoracic+mediastinum	1	3.85%

## Data Availability

The datasets used during the present study are available from the corresponding author upon reasonable request.

## Supplementary Materials

Supplementary Materials Table 1s. Globin mutations identified in 52 NTDT patients. Table 2s. Globin genotypes identified in 52 NTDT patients. Table 3s. The distribution of blood transfusion requirement in 52 NTDT patients. Table 4s. The distribution of iron chelation therapy in 52 NTDT patients.

## References

[1] Musallam K. M., Rivella S., Vichinsky E., Rachmilewitz E. A. (2013). Non-transfusion-dependent thalassemias. *Haematologica*.

[2] Taher A. T., Musallam K. M., Karimi M. (2010). Overview on practices in thalassemia intermedia management aiming for lowering complication rates across a region of endemicity: the optimal care study. *Blood*.

[3] Taher A., Isma'eel H., Cappellini M. D. (2006). Thalassemia intermedia: revisited. *Blood Cells, Molecules, and Diseases*.

[4] Haidar R., Mhaidli H., Taher A. T. (2010). Paraspinal extramedullary hematopoiesis in patients with thalassemia intermedia. *European Spine Journal*.

[5] Musallam K. M., Taher A. T., Duca L., Cesaretti C., Halawi R., Cappellini M. D. (2011). Levels of growth differentiation factor-15 are high and correlate with clinical severity in transfusion-independent patients with beta thalassemia intermedia. *Blood Cells, Molecules, and Diseases*.

[6] Gardenghi S., Grady R. W., Rivella S. (2010). Anemia, ineffective erythropoiesis, and hepcidin: interacting factors in abnormal iron metabolism leading to iron overload in *β*-thalassemia. *Hematology/Oncology Clinics of North America*.

[7] Ricchi P., Ammirabile M., Costantini S. (2012). A useful relationship between the presence of extramedullary erythropoeisis and the level of the soluble form of the transferrin receptor in a large cohort of adult patients with thalassemia intermedia: A prospective study. *Annals of Hematology*.

[8] Kautz L., Jung G., Valore E. V., Rivella S., Nemeth E., Ganz T. (2014). Identification of erythroferrone as an erythroid regulator of iron metabolism. *Nature Genetics*.

[9] Arezes J., Foy N., McHugh K. (2018). Erythroferrone inhibits the induction of hepcidin by BMP6. *Blood*.

[10] Schotten N., Laarakkers C. M. M., Roelofs R. W., Origa R., van Kraaij M. G. J., Swinkels D. W. (2017). EPO and hepcidin plasma concentrations in blood donors and *β*-thalassemia intermedia are not related to commercially tested plasma ERFE concentrations. *American Journal of Hematology*.

[11] Ganz T., Jung G., Naeim A. (2017). Immunoassay for human serum erythroferrone. *Blood*.

[12] Yang G., Liu R., Peng P. (2014). How Early Can Myocardial Iron Overload Occur in Beta Thalassemia Major?. *PLoS ONE*.

[13] Ricchi P., Meloni A., Spasiano A. (2015). Extramedullary hematopoiesis is associated with lower cardiac iron loading in chronically transfused thalassemia patients. *American Journal of Hematology*.

[14] Taher A. T., Musallam K. M., El-Beshlawy A., etal. (2010). Age-related complications in treatment-naive patients with thalassaemia intermedia. *British Journal of Haematology*.

[15] Ginzburg Y., Rivella S. (2011). *β*-thalassemia: A model for elucidating the dynamic regulation of ineffective erythropoiesis and iron metabolism. *Blood*.

[16] Origa R., Cazzola M., Mereu E. (2015). Differences in the erythropoiesis-hepcidin-iron store axis between hemoglobin H disease and *β*-thalassemia intermedia. *Haematologica*.

[17] Tantawy A. A. G., Adly A. A. M., Ismail E. A. R., Youssef O. I., Ali M. E. (2015). Growth differentiation factor-15 in children and adolescents with thalassemia intermedia: Relation to subclinical atherosclerosis and pulmonary vasculopathy. *Blood Cells, Molecules, and Diseases*.

[18] Tanno T., Bhanu N. V., Oneal P. A. (2007). High levels of GDF15 in thalassemia suppress expression of the iron regulatory protein hepcidin. *Nature Medicine*.

[19] Fertrin K. Y., Lanaro C., Franco-Penteado C. F. (2014). Erythropoiesis-driven regulation of hepcidin in human red cell disorders is better reflected through concentrations of soluble transferrin receptor rather than growth differentiation factor 15. *American Journal of Hematology*.

[20] Tanno T., Porayette P., Sripichai O. (2009). Identification of TWSG1 as a second novel erythroid regulator of hepcidin expression in murine and human cells. *Blood*.

[21] Theurl I., Finkenstedt A., Schroll A. (2010). Growth differentiation factor 15 in anaemia of chronic disease, iron deficiency anaemia and mixed type anaemia. *British Journal of Haematology*.

[22] Kautz L., Jung G., Du X. (2015). Erythroferrone contributes to hepcidin suppression and iron overload in a mouse model of *β*-thalassemia. *Blood*.

[23] Honda H., Kobayashi Y., Onuma S. (2016). Associations among erythroferrone and biomarkers of erythropoiesis and iron metabolism, and treatment with long-term erythropoiesis-stimulating agents in patients on hemodialysis. *PLoS ONE*.

[24] Ricchi P., Ammirabile M., Spasiano A. (2014). Extramedullary haematopoiesis correlates with genotype and absence of cardiac iron overload in polytransfused adults with thalassaemia. *Blood Transfusion*.

[25] Sousos N., Adamidou D., Klonizakis P. (2017). Presence of the IVS-I-6-Mutated Allele in Beta-Thalassemia Major Patients Correlates with Extramedullary Hematopoiesis Incidence. *Acta Haematologica*.

[26] Shin K. H., Sharma S., Gregoritch S. J., Lifeso R. M., Bettigole R., Yoon S. S. (1987). Combined radiotherapeutic and surgical management of a spinal cord compression by extramedullary hematopoiesis in a patient with hemoglobin e beta-thalassemia. *Acta Haematologica*.

[27] Vichinsky E. (2016). Non-transfusion-dependent thalassemia and thalassemia intermedia: Epidemiology, complications, and management. *Current Medical Research and Opinion*.

[28] Belhoul K. M., Bakir M. L., Saned M.-S., Kadhim A. M. A., Musallam K. M., Taher A. T. (2012). Serum ferritin levels and endocrinopathy in medically treated patients with *β* thalassemia major. *Annals of Hematology*.

