# Supplementary material for: Erythropoiesis and Iron Homeostasis in Non-Transfusion-Dependent Thalassemia Patients with Extramedullary Hematopoiesis
Source: Biomed Res Int. 2019 Jan 30;2019:4504302. doi: 10.1155/2019/4504302 (PMC6374788; doi:10.1155/2019/4504302)
Supplement: Supplementary Materials — Table 1s. Globin mutations identified in 52 NTDT patients. Table 2s. Globin genotypes identified in 52 NTDT patients. Table 3s. The distribution of blood transfusion requirement in 52 NTDT patients. Table 4s. The distribution of iron chelation therapy in 52 NTDT patients. [file 4504302.f1.docx]

| **Table 1s.Globin mutations identified in 52 NTDT patients** | | | | | |
| --- | --- | --- | --- | --- | --- |
| **Mutation** | **HGVS nomenclature** | **Type of thalassemia** | **EMH(+)** | **EMH(-)** | **p value** |
| **CD 26 (G->A)** | HBB:c.79G>A | **HbE** | **7** | **7** | 0.565 |
| **17 (A->T)** | HBB:c.52A>T | **β^0^** | **6** | **2** |  |
| **41-42(-TTCT)** | HBB:c.126_129delCTTT | **β^0^** | **5** | **5** |  |
| **-28 (A->G)** | HBB:c.-78A>G | **β+** | **3** | **4** |  |
| **IVS-II-654** | HBB:c.316-197C>T | **β+** | **1** | **1** |  |
| **IVS-I-1** |  | **β^0^** | **1** | **0** |  |
| **71/72 (+A)** | HBB:c.216_217insA | **β^0^** | **1** | **0** |  |
| **-29 (A->G)** | HBB:c.-79A>G | **β+** | **1** | **0** |  |
| **IVS-II-5** | HBB:c.315+5G>C | **β+** | **0** | **2** |  |
| **27/28 (+C)** | HBB:c.84_85insC | **β^0^** | **0** | **2** |  |
| **Hb CS** |  | **Hb variants** | **8** | **7** |  |
| **Hb Youngstown** | HBB:c.305A>C | **Hb variants** | 1 | **2** |  |
| **Hb Broomhill** |  | **Hb variants** | **0** | **1** |  |
| **Hb QS** |  | **Hb variants** | **0** | **1** |  |

| **Table 2s. Globin genotypes identified in 52 NTDT patients** | | | |
| --- | --- | --- | --- |
| **Globin**  **genotype** | **EMH(+)** | **EMH(-)** | **p value** |
|  |  |  | 0.957 |
| **β+/β+** | 4 | 4 |  |
| **β/E** | 7 | 7 |  |
| **β0/β0** | 4 | 2 |  |
| **β+/β0** | 1 | 3 |  |
| **HbH** | 9 | 8 |  |
| **Hb Youngstown** | 1 | 2 |  |

| **Table 3s. The distribution of blood transfusion requirement in 52 NTDT patients** | | | | |
| --- | --- | --- | --- | --- |
| **Group** | **EMH+** | **EMH-** | **X^2^** | **p value** |
| **Rare** | 18 | 15 | 5.44 | 0.082 |
| **Occasional** | 9 | 6 |  |  |
| **Regular** | 0 | 5 |  |  |

| **Table 4s The distribution of iron chelation therapy in 52 NTDT patients** | | | | |
| --- | --- | --- | --- | --- |
| **Group** | **EMH+** | **EMH-** | **X^2^** | **p value** |
| **Yes** | 6 | 7 | 0.103 | 0.749 |
| **No** | 20 | 19 |  |  |
